# Innovative micro physiological systems for vaccine development

**DOI:** 10.1080/21645515.2025.2518641

**Published:** 2025-06-17

**Authors:** Kendra Reynaud, Scott Atwell, Christophe Védrine, Cyril Guyard, Laurent Beloeil

**Affiliations:** BIOASTER Technology Research Institute, Lyon, France

**Keywords:** Micro physiological systems, biomimetic, translational, pre-clinical, vaccine, reactogenicity, immunogenicity, *in vitro*

## Abstract

Micro Physiological systems (MPS) are biomimetic platforms that reconstitute the complex architecture of human organs and tissues *in vitro*. MPSs have the capacity to improve clinical development pipelines as they address certain translational limitations that exist in other pre-clinical models by providing human-relevant microenvironments. These platforms have already demonstrated their potential in vaccine development, as several variations of MPSs have recently been published that provided results relevant to vaccine trials in humans. Previous MPSs include approximations of peripheral and lymphoid tissue in static platforms to study the innate and adaptive response to vaccination, as well as a lymphoid-follicle-on-chip under dynamic flow which produces antigen-specific antibodies to vaccination. Up-and-coming devices could be used to study the interplay between vaccine reactogenicity and innate immune stimulation, and thereby derisk vaccine targets by elucidating their inflammatory profiles prior to advancement to clinical trials.

## Introduction to micro physiological systems

Micro Physiological Systems (MPS), often referred to as “organs-on-chips,” are advanced biomimetic *in vitro* platforms that reconstitute the structure, function, and physiological responses of human organs and tissues.^1^ By integrating microfluidics, tissue engineering, and biosensing technologies, MPSs replicate the complex microenvironment of human organs, including tissue-tissue interfaces, mechanical forces, and biochemical gradients.^2^

**Figure 1.**
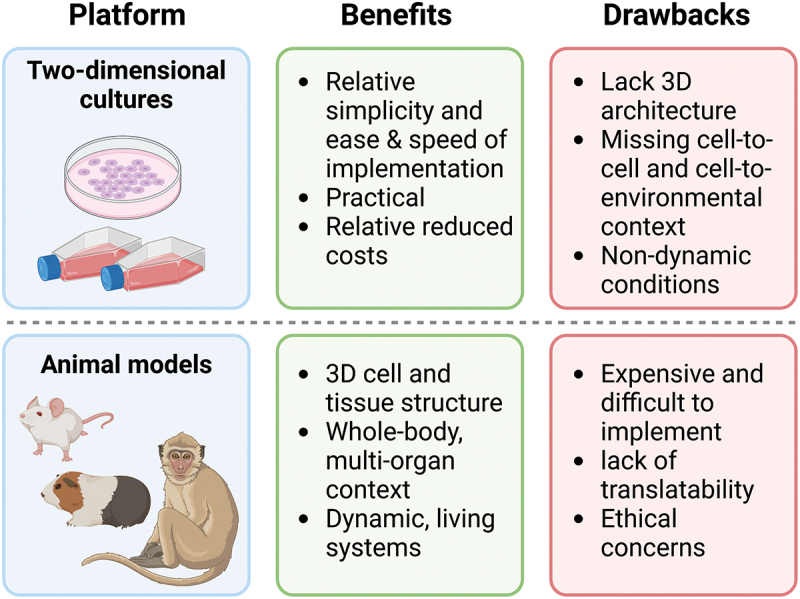
The advantages and disadvantages of current preclinical models.

The development of MPSs is driven by the need for more predictive models in drug discovery, toxicology, and disease research (Figure 1). Traditional two-dimensional (2D) systems, including whole-blood and cell co-culture assays, are often utilized for their relative simplicity, speed of implementation and practicality.^3^ While 2D systems have offered valuable insights, such as revealing the functions of human leukocytes, they fall short in accurately representing the *in vivo* immune environment. This limitation arises because they do not replicate the three-dimensional structure and dynamic conditions found in living tissues *in vivo* .^4^ Animal models overcome these challenges, yet they often fail to accurately replicate human physiology due to species-specific differences.^5^ Indeed, while there is no exact percentage breakdown for species-specific translatability issues, it is estimated that around 30% of drug failures in clinical trials may be linked to limitations of preclinical models, including divergence of animal models from human responses. These differences can affect pharmacokinetics, pharmacodynamics, and disease mechanisms, leading to unexpected outcomes when drugs are later tested in humans.^6,7^ MPSs bridge this gap by providing a human-relevant platform that can simulate organ-level responses to drugs, toxins, and/or pathogens. MPSs also usually provide better access to real-time monitoring of cellular responses to stimuli than *in-vivo* models.^8^ Finally, MPSs address the Three R’s principle that attempts to reduce our over-reliance on animal research, namely “Replacement, Reduction and Refinement,”^9^ by providing a strong alternative to animal models that ameliorates ethical concerns around animal testing while providing the added advantage of human-specific data.^10^

## The development of MPSs

The first published organ-on-chip platform contained the key structural, functional and mechanical properties of the human alveolar-capillary interface, comprising the fundamental functional unit of the lung.^11^ The lung-on-a-chip demonstrated its potential by recapitulating certain organ-level functions such as responding to proinflammatory mediators with activation of the microvascular endothelium as well as transmigration of neutrophils from the microvasculature to the alveolar compartment, a hallmark of lung inflammation.^11,12^ This study demonstrated the ability of *in vitro* platforms to model physiological behaviors and immune responses of the human lung, thereby laying the foundation for the development of other organ-on-chip platforms spanning a spectrum of organ types, including the liver, lung, gut, kidney, brain, heart and skin.^2,13^ While MPSs can recapitulate single organ properties, studying certain disease states or drug testing regimes requires interactions with multiple organs in the body. There is thus increasing interest in linking different tissue compartment MPSs to better recapitulate drug uptake, circulation and biotransformation throughout the body.^14^ There are various “body-on-a-chip” platforms which have subsequently emerged, enabling researchers to study multi-organ interactions and systemic effects.^15^ These systems have the potential to recapitulate both drug efficacy and potential toxicity in different organs.^14^

## Challenges entailed in the implementation of MPSs

While MPSs offer several distinct advantages over alternative methods available to researchers, there are also certain disadvantages that must be acknowledged. These include a lack of standardization in the design and implementation of these platforms, which can hinder the reproducibility of experiments and results.^16^ Such variability can arise from parameters such as the balance between organ sizes versus the other components in the system, inter-organ transportation rates in multi-organ systems, as well as liquid-to-cell ratios.^14^ Indeed, replicating physiological conditions *in vitro* is very challenging, and often trade-offs between modeling *in vivo* processes and experimental success must be made.^16^ Cell sourcing also presents a certain challenge, as immortalized cell lines provide a near unlimited source of cells that are extensively validated with reproducible culture protocols, yet they are only one approximation of a primary cell phenotype. Primary cells offer higher physiological relevance, yet can be difficult to source, particularly for certain tissue types such as neural cells, and they are subject to batch and donor variation.^17^ Induced pluripotent stem cells (iPSC) overcome some of the above drawbacks in that they are patient-derived and hold the potential to form different tissues in the body but still have the disadvantage that the tissue resulting from iPSC differentiation usually presents an immature phenotype.^18^

The construction of MPSs also presents a challenge since the engineers who have the knowledge required to build a functional organ-on-chip must also understand the complex *in vivo* biomechanical and environmental stimuli necessary to properly recapitulate tissue- and organ-level functions.^19^ Certain limitations are also inherent in the choice of materials used to make the MPS. Polydimethylsiloxane (PDMS), the material of choice for most microfluidics-based-devices, has certain drawbacks, namely the adsorption of hydrophobic compounds and leaching of un-crosslinked oligomers, which present a major obstacle for drug discovery assays.^20^ Developing alternatives to PDMS have therefore been a recent subject of interest.^21,22^ Given the inherent limitations of each material, it is crucial for users to leverage biomaterial science to determine which material best fits their specific purposes.

Despite the current roadblocks standing between MPSs and their widescale-adoption into drug-discovery programs, they offer major advantages that make these platforms worth pursuing. They offer translational relevance to human physiology and pathophysiology that currently implemented 2D methods or animal models cannot provide.^23^ In addition, they offer potential for personalized medicine in that these systems can be tailored to study variations in an individual or a particular cohort’s responses to prophylactics, therapeutics and treatment regimens that other methods cannot offer. There is therefore belief in the field that we are at the “tipping point” where the possibility of real reduction in the use of animal models with the application of MPSs in drug testing is more realistic than ever.^13^

## Regulatory positioning on the implementation of MPSs in clinical development

Recently, the U.S. Food and Drug Administration (FDA) announced a roadmap to phase out animal testing in preclinical safety and toxicity studies over the next three-to-five years.^24^ This roadmap builds upon the FDA Modernization Act 2.0, voted on in 2022, and allows the agency to accept New Approach Methodologies (NAMs) such as organ-on-chip and MPS as a replacement to animal studies in new drug applications. The FDA Modernization Act 3.0, introduced in early 2024 and expected to pass during 2025, further instructs the FDA to establish processes to qualify NAMs within a two-year timeline. This Act also tasks the agency to expedite the review of applications using qualified NAMs, thus incentivizing sponsors.^25^ Similarly, the European Medicines Agency (EMA) introduced guidelines in 2017 to encourage the development and regulatory acceptance of NAMs.^26^ Multiple European initiatives, such as the Organ on Chip In Development Project (ORCHID) and the Europe Oran-on-Chip Society (EUROoCS) have formed in order to foster organ-on-chip development, standardization, and regulatory acceptance.^27,28^ So far, promising developments have begun to emerge, as exemplified by the European Parliament calling for an EU-wide action plan to accelerate the shift away from animal testing. The European Commission roadmap is currently underway,^29^ and such initiatives demonstrate the high interest in developing new animal-free technologies, from an ethical standpoint as well as an economic and a medically relevant perspective.

## Adapting MPSs to vaccine research and development

One field where MPSs offer great potential is the field of vaccine research and development. The importance of vaccines in combating infectious diseases is difficult to overstate. Safe and effective vaccines have led to a drastic reduction in death and morbidity from communicable diseases in the 20th and 21st centuries.^30^ The recent COVID-19 pandemic, where up to 18 million deaths were averted through vaccination, highlights the need for the ongoing development of novel vaccines to counter emerging threats.^31^ However, vaccine development is a long-term investment fraught with technical challenges and major expenses. Few candidates make it past the initial hurdles in early clinical development due to the large and very expensive efficacy trials.^32^

A primary issue with the vaccine development pipeline is the lack of translatability between assays in preclinical animal models and the human immune system.^33^ Indeed, animal models have had a low success rate in predicting vaccine effectiveness in humans,^34^ even in models closest to humans such as non-human primates. For example, an Adenovirus type 5 (Ad5)-based vaccine for Simian immunodeficiency virus (SIV) caused a substantial reduction in viral load and preservation of CD4+ T cell count in Macaques yet failed to prevent either HIV-1 infections or suppress viral load in subsequently infected human subjects in a human Phase 2B efficacy trial.^35^ Over-reliance on animal studies has generated ethical concerns around the harm versus the benefit of such studies,^36^ not to mention there are increasing concerns around the limited supply of non-human primates for global research.^37^ There is therefore great interest in reducing the reliance of vaccine research on animal testing by establishing advanced *in vitro* models based on human cells.^38^

## Human immune response to vaccination in model in vitro systems

*In vitro* models can be used to study the response to vaccines in various ways, either by benchmarking the immune response or gaining mechanistic insights to vaccine-induced immunity at the cellular and molecular level. These models could also be used to study vaccine efficacy and safety, or to develop personalized vaccine models. Evaluation of vaccine efficacy in an MPS could be achieved by measuring the production of a known correlate of protection, or a surrogate of protection if this information is available, against a given pathogen.^39^ An alternate possibility would be to develop the concept of a “challenge-on-chip,” mimicking conventional animal challenge studies. In the challenge-on-chip, immune cells that have been primed with a vaccine would be transferred into an MPS and subsequently challenged with a corresponding pathogen to assess their ability to eradicate or neutralize that pathogen. While this technology, to our knowledge, does not yet exist for vaccine development, measuring the immune response “on chip” has been demonstrated in an MPS for cancer immunotherapy validation.^40^ Vaccine safety could be assessed on chip by benchmarking blood donors’ responses to new vaccines with their responses to already licensed vaccines with known acceptable safety profiles, thereby ranking these new vaccines within a previously-defined tolerability zone.^41–43^

In certain instances, a simpler 2D *in vitro* method is sufficient and appropriate for studying the immune response to a vaccine. Whole blood assays have been used to benchmark various adjuvant formulations, including the age-and adjuvant-specific production of cytokines and chemokines post-stimulation.^44,45^ Likewise, monocyte-derived dendritic cells (MoDCs) cultured with various toll-like receptor (TLR) agonists or with whole-cell pertussis vaccine were capable of producing biomarkers, such as prostaglandin E2 and IL-1β, that match the relative reactogenicity produced in humans *in vivo*.^46,47^ However, as mentioned above, 2D systems are missing important elements that are key in driving immune responses *in vitro*, including cell-to-cell interactions and other architectural features fundamental to the cellular environment, which are particularly important when a vaccine’s mechanism of action is ill-defined. Other recent reviews have done an excellent job recapitulating all the latest *in vitro* models for studying vaccines,^3,48,49^ therefore we will not aim to do so here. Instead, we will discuss a few key examples that highlight the potential in the field.

## The MIMIC platform

A foundational *in vitro* platform for studying vaccine responses is the Modular Immune *In vitro* Construct (MIMIC), which is a static biomimetic model of the human immune system. The MIMIC platform comprises three distinct modules, the Peripheral Tissue Equivalent (PTE), the Lymphoid Tissue Equivalent (LTE), and the Functional Assay construct, each of which are designed to represent different aspects of the immune response to vaccines.^50,51^ The PTE module was designed to represent innate immunity that occurs in peripheral tissues. It is a 3D tissue engineered construct that is composed of a confluent human endothelial monolayer over an extracellular collagen matrix.^52^ Purified peripheral blood mononuclear cells (PBMCs) are placed on top of the model, and an immature monocyte population extravasates across the endothelial layer into the matrix. Within the matrix, the monocytes spontaneously differentiate into dendritic cells (DCs) and later reverse transmigrate, resembling the entrance of DCs into the lymphatic system.^53,54^ Indeed, the kinetics of the DC migration and differentiation within the MIMIC module largely matches *in vivo* data in humans and animals.^55,56^ These migratory DCs remain largely immature in the absence of stimulation, yet they are capable of maturing and processing antigen when stimulated by adjuvants, and are even capable of initiating antigen-specific immune responses in downstream coculture assays in the LTE that mimic the adaptive immune system in the lymph node.^53^ The MIMIC platform has been tested with a variety of monoclonal antibodies and vaccines, including HBV, Influenza, Tetanus,^50^ Meningitis B, and Yellow Fever,^57^ to show that MIMIC can generate responses consistent with known *in vivo* immune profiles.^58^ These responses include serum antibody responses,^50^ upregulation of cytokines, expression of transcription factors and other vaccine-relevant genes,^57^ and induction of specific CD4+ and CD8+ T cell responses.^59^ Indeed, the MIMIC system has even been able to recapitulate the influence of immune senescence on the anti-trivalent influenza vaccine responses of aging populations via reduction of total and functional IgG, and impairment in the generation of antigen-specific CD4+ T cells.^51^ The MIMIC platform does entail certain limitations, including, but not limited to, a focus mainly on peripheral blood mononuclear cells (PBMCs) to the exclusion of other important immune cells, for example neutrophils or certain tissue-resident cells. It is also a relatively simplified system in that it lacks tissue complexity. Despite these drawbacks, the success of the MIMIC platform offers a way to obtain clinically relevant data on the immunostimulatory potential of vaccines or other immune modulators at the preclinical level to improve the outcomes of later clinical trials.^51^

## Age-specific human tissue constructs

Another important system in the field are age-specific human tissue constructs (HTCs) which reproduce age- and antigen-specific responses of human cohorts *in vivo* .^60^ The primary difference between the HTCs and previous similar devices such as MIMIC is derived from the use of exclusively endotoxin-free human biomaterials, and in particular use of autologous humoral and cellular components which more accurately comprise human microphysiology and ontogeny.^60^ These HTCs contain monocytes from either newborn or adult donors which autonomously extravasate within the tissue constructs (TCs) across a layer of endothelial cells and a layer of human extracellular matrix (ECM) proteins into a basement cushion of human collagen within a static environment. After the removal of non-extravasated monocytes, the TCs are either treated for 48 hours with autologous plasma alone (unstimulated), or autologous plasma containing either adjuvant or vaccine formulations (stimulated), and then reverse-transmigrated cells are harvested and analyzed.^46,60^ In the absence of stimulation, reverse-transmigrated cells are primarily monocyte- or macrophage-like, whereas stimulated cells present a significantly more dendritic cell (DC)-like phenotype.^46,60^ Notably, the number of mature DCs is higher in adults than newborns after stimulation with most adjuvants and the BCG vaccine, which is expected as human newborn leukocytes are known to respond weakly to certain adjuvants.^60^ Interestingly, substitution of newborn plasma with adult plasma enhanced the adjuvant-induced maturation of newborn DCs, which is consistent with the previously reported immunosuppressive effects of newborn plasma toward TLR-mediated pro-inflammatory signaling.^61^ These TC-derived DCs were also capable of stimulating antigen-specific responses in autologous naïve newborn CD4+ T cells after *in vitro* immunization with BCG vaccine.^60^ The capacity to stimulate such a response is an important achievement for an *in vitro* system, as the T cell memory response is crucial for the adaptive immunity required to limit or prevent viral reinfection, and indeed many vaccine regimens target robust T cell memory responses.^62^ There are also limitations to the age-specific HTS which are similar to those of the MIMIC system mentioned above. However, the ability to recapitulate key aspects of neonatal immune ontogeny, which is quantitatively and qualitatively different to that of the adult, is a valuable tool in developing more effective vaccines for newborns, in particular as they are at high risk of infection and also receive the greatest number or vaccines.^63^

## Lymph node on chip

The lymph node (LN) on chip is a recent major advance in MPS technology that has great potential in the field of vaccine research. The LN is difficult to recapitulate *in vitro*, as it is an architecturally complex organ composed of various immune cell types that are spatially divided into different micro-domains.^64^ LNs, together with the other secondary lymphoid organs, including the spleen and Peyer’s patches (PPs), perform immune surveillance throughout the body.^65^ While the spleen and PPs act as sentinels for harmful materials in the blood and gastrointestinal tract, respectively, LNs are situated in proximity to lymphatic and vascular branching points.^66^ At these strategic junctions, they detect foreign particles and antigens circulating within the lymphatic fluid and facilitate immune cell entry from surrounding tissues.^67^ Lymphoid follicles (LFs) are a specialized tissue microenvironment that are responsible for generating an adaptive immune response.^68^ LFs normally exist within the cortex of secondary lymphoid organs, and they can also form ectopically at the site of inflammation in impacted tissues during chronic inflammation, or after immunization or infection.^69^

Various groups have developed lymph node-on-chip platforms to mimic the natural LN microenvironment and associated immune functions under the dynamics of lymphatic flow.^64,70,71^ Here, we will focus primarily on the platform most relevant to vaccine research: the LF-on-chip that has been used to study seasonal influenza vaccination responses.^68^ In contrast to the two earlier devices, it is a microfluidic device that contains two channels separated by a porous membrane. In the lower chamber reside B and T cells from a human donor cultured at a high density within a 3D extracellular matrix gel containing type I collagen. DCs differentiated from autologous monocytes are introduced into the B and T cell compartment as 2% of the population to recapitulate DC antigen presentation in the draining lymph node.^68^ Continuous perfusion of culture medium supplies oxygen in the parallel upper channel.^68^ The dynamic flow provided through this upper chamber is required for the spontaneous self-assembly of T and B cells into larger, more numerous 3D aggregates, and to prevent the autoactivation of B cells.^72^

As a demonstration of its usefulness in vaccine development and adjuvant testing, the LF-on-chip was challenged with split H5N1 seasonal influenza antigen with and without squalene-in-water emulsion (SWE) adjuvant. The protective immunity induced by vaccination requires production of antigen-specific antibodies by plasma cells, including those in ectopic LFs.^73^ The LF-on-chip had increased levels of anti-HA antibody secretion relative to 2D cultures, which were enhanced by the addition of SWE, and this was accompanied by increases in LF size and number. When inoculated with the trivalent Fluzone influenza vaccine, the LF-on-chip formed plasma cells and produced anti-Hemagglutinin IgG antibodies, as well as secreted cytokines over similar timescales to vaccinated humans.^68,74,75^ In addition, among the 8 donor samples challenged with Fluzone, the LF-on-chip was able to detect donor-specific responses, including variable levels of antibodies and cytokines produced in high- versus low-responding donors. Taken together, this device has demonstrated its utility as a human-relevant preclinical tool for assessing 3D follicle and plasma cell formation, expression of cytokines, and antibody generation in response to vaccination. Finally, as is the case with other *in vitro* platforms, there are also limitations with LF-on-chip, namely it contains an over-simplified geminal center microarchitecture compared to what exists in lymphoid follicles *in vivo*, as well as other technical drawbacks including limited real-time imaging and small sample volumes, making it challenging to measure low-abundance molecules.^76^ Despite these drawbacks, LF-on-chip is a major contribution to the field, notably because prior lymph node-on-chip platforms had experimental timelines on the order of hours, whereas LF-on-chip is usable for many days up to *weeks* at a time.^76^

## In vitro prediction of innate immune response to vaccination

**Figure 2.**
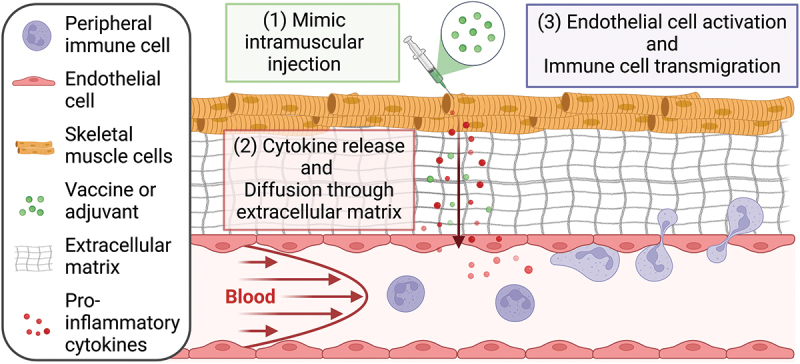
An MPS with muscle fibers and a dynamic, endothelial-cell-lined blood flow compartment that recapitulates the innate immune response to vaccination: (1) the vaccine is introduced to the skeletal muscle compartment, (2) inflammatory mediators diffuse across the ECM into the blood compartment where they (3) stimulate the endothelial and immune cells and induce immune cell migration across the endothelial barrier.

The aforementioned platforms have distinguished themselves in benchmarking the immune response to vaccination and recapitulating key functions of the adaptive immune response. However, an MPS that contains the elements of the local vaccine delivery site is essential for understanding the early steps in generating immune responsiveness, as the magnitude of local innate immune responses starting at the vaccine delivery site initially controls the subsequent adaptive immune responses.^77^ Indeed, intramuscular (IM) injections are the preferred route of delivery for vaccinations due to a lower risk of serious adverse reactions^78,79^ and generating more robust humoral responses.^80^ Therefore, to recapitulate local reactions at the intramuscular vaccine injection site, a dynamic organ-on-chip platform that includes the muscle-to-blood interface is needed. Such a platform is currently under development at BIOASTER (Figure 2). The design of this platform will enable the prediction of innate immune responses in the muscle compartment that are known to initially control subsequent adaptive immune responses.^81–83^

To emulate the complex, highly vascularized network of muscle fibers and endothelial and immune cells in muscle tissue,^84,85^ our platform includes skeletal muscle cells in addition to the standard endothelial and immune cells. The inclusion of muscle cells is essential to better recapitulate *in vivo* processes, as vaccine adjuvants administered to the muscle induce transient, local inflammation, which promotes the diffusion of inflammatory mediators.^77^ This inflammation, in turn, promotes vaccine antigen uptake by immune cells and subsequent migration of vaccine loaded cells to the draining lymph nodes.^86^ Both the muscle and endothelial tissues of this platform are derived from primary cell cultures, allowing for greater physiological relevance while also being widely available for the purpose of standardization. Neutrophils are also included in addition of the traditional PBMCs^51,87^ as they have emerged as an important nonredundant regulator of innate and adaptive immunity, influencing the activity of other cells of the immune system.^77^ Importantly, a dynamic circulatory system is included in the platform to mimic a unidirectional and physiologically relevant shear stress of the endothelial and recirculating immune cells in the blood vessel compartment under controlled oxygen tension. Through the application of shear stress, the goal is to better recapitulate the physiological phenotypes of endothelial cells, which otherwise highly express cytokines and other inflammatory proteins under reduced or perturbed flow conditions.^88,89^

This platform is compatible with a diversity of longitudinal and end-point readouts, including classical immunology assays as well as transcriptomics, proteomics, and single-cell techniques. Each sampling removes less than 5% of circulating cells and media to achieve minimal disruption of the system while allowing for days-long time course experiments. A special effort has also been made to ensure that the platform is compatible with live microscopy to enable real-time, noninvasive monitoring of the system using optical means, including high-resolution and live microscopy.

The limitations of this device include our choice of sourcing of both the muscle and endothelial tissues from primary cell cultures; although this adds the benefit of greater physiological relevance in our assays, it also entails the drawbacks of batch and donor variation.^90^ Our current iteration of this platform also lacks certain tissue-resident immune cells, particularly DCs, which is one disadvantage of using fresh PBMCs in our system as this does not allow for timelines that could enable autologous DC enrichment. Finally, this platform is best suited to studying the innate immune response to vaccination, whereas a future generation of the platform will need to be coupled with an LN-on-chip-like system to also study the adaptive immune response to vaccination. Despite these limitations, this platform promises a unique opportunity to evaluate the innate immune response to preclinical vaccine candidates, as well as enabling the downstream selection of targets that elicit a longer-lasting adaptive immune response. In addition, a “personalized medicine” approach will also be possible through patient specificity in this platform by sourcing primary immune cells from different blood donors. The potential to use the device to develop therapeutics for subgroups with certain diseases or comorbidities, or to select drugs that perform best for a given individual, could be revolutionary.

## Reactogenicity: clinically well-defined yet still a mechanistic conundrum

In the field of vaccine development, certain outstanding questions have yet to be fully resolved. Chief among these is to what extent the body’s physical reaction to a vaccine is related to the innate immune response, which is known to control the magnitude and quality of the adaptive immune response.^81,91^ It is thought that a certain amount of inflammation is required to trigger an appropriate adaptive response to vaccination; indeed highly immunogenic vaccines often trigger more adverse effects than lowly immunogenic ones.^92,93^ For instance, a recent study with the mRNA COVID-19 vaccine found a significant correlation between increased post-vaccination symptoms such as pain, fever and swelling, and anti-SARS-CoV-2 antibody titers. However, too strong an inflammatory response can be dangerous to the patient and could lead to negative perceptions of the vaccine amongst the target population.^94^ Indeed, while vaccines can protect us from infectious diseases by preparing the immune system to produce antibody- or cell-mediated responses against a pathogen, they can also cause adverse and harmful effects.^95^ Thus, a successful vaccine candidate has to trigger sufficient inflammation and immunogenicity, yet this must be at a tolerable level for the patient and safe for a general population.^94,95^ The field of vaccine development is therefore actively pursuing a better understanding of the connection between improved immune responses and the transient physical reactions to a vaccine, also known as reactogenicity.

In humans, reactogenicity can be measured by local symptoms around the site of injection, including pain, redness, swelling and induration, and systemic symptoms such as fever, myalgia, headache or rash.^94^ While some of these reactions can be measured objectively, such as fever or swelling, others rely on inherently subjective perspectives of the patient (e.g. headache or fatigue).^95,96^ The subjectivity of symptom reporting and variations in patients’ lifestyles and environments can make it challenging to untangle which signs are caused directly by vaccination versus other confounding factors.^94,96^ In addition, not all vaccines cause the same reactogenic symptoms, and not all vaccines report experiencing symptoms, yet in these cases there is usually still a sufficient immune response induced post-vaccination.^97^ The interface between clinical phenotypes and molecular mediators of inflammation and adaptive immunity is not simple to elucidate, making reactogenicity difficult to study on the level of the mechanism of action. Likewise, it can be difficult to determine whether what is measured at the molecular level will translate into a clinical effect or phenotype. Therefore, recent efforts have been put forth to identify molecular biomarkers that are a more reliable indicator of immunogenicity or vaccine-induced protection than the presence or intensity of reactogenicity symptoms.^97–100^

To date, no single biomarker predictive of systemic reactogenicity across different vaccines has been identified in human clinical studies. Rather, a composite of biomarkers that include the interferon-signaling pathway, C-reactive protein, and interleukin 6 were activated in vaccines that enhance the adaptive immune response.^98,99^ The availability of adapted *in vitro* platforms would contribute to a better understanding of the molecular biomarkers produced post-vaccination, and which of these actually correlate with better longer term immune protection. Once established, MPS platforms could then provide an accurate prediction of vaccine reactogenicity in humans and facilitate the screening and early prioritization of successful new vaccine candidates.^3,3,46^

## Studying reactogenicity with in vitro platforms

As a proof-of-principle, a recent study paired the MIMIC PTE module to evaluate vaccine innate immune profiles coupled with machine learning to model vaccine reactogenicity in a clinical setting. They treated PBMCs from 40 healthy donors with 10 different vaccines with varying reactogenicity in the MIMIC PTE platform. Cells and supernatants from these assays were then analyzed with flow cytometry and chemokine/cytokine assays. In the same study, a dataset of adverse events from publicly available clinical trial data was assembled to create a reactogenicity scoring framework for the vaccines. A machine learning approach was used to predict the incidence of clinical adverse events from the aforementioned *in vitro* assay data to demonstrate that the relative levels of IL-1B, IL-6, IL-10, and CCL4 have higher predictive importance for adverse event risk.^101^ This landmark study highlights the contributions that MPSs could make in vaccine development, especially in assessing the risk of adverse events of novel vaccine candidates. However, the study was limited by certain experimental factors, including the use of a pre-defined set of chemokines and cytokines that were measured within a relatively short 48-hour timescale. Additionally, the assay was performed in the MIMIC model, which does not have a muscle compartment or dynamic flow, the importance of which has already been discussed above. Therefore, a more longitudinal approach with an unbiased readout in an MPS that recapitulates the physiological relevance of the site of injection could provide a more comprehensive understanding of the biomarkers with predictive value for clinical adverse events. Such a study would need to include a sufficient number of donors to capture the range of variability between individuals, for instance the aforementioned MIMIC model study used 40 donors to evaluate relevant biomarker production.^101^ Indeed, MPS technology allows for the massive parallelization of the devices to increase statistical robustness and ensure a sufficient depth of subjects tested. Finally, such a study would not be intended to replace a clinical Phase I study but would instead provide key information on vaccine performance on specific populations prior to testing in humans.

## Discussion

Micro Physiological systems have demonstrated their utility in the development of novel therapeutics^10,38^ and in particular vaccines.^101^ This is in large part due to a better representation of human physiology than 2D assays or even animal models, which have limited translatability to the human immune response.^5^ Despite the limitations of *in vitro* platforms, the data thus far approximating human vaccine reactogenicity and immunogenicity within an MPS are highly encouraging.^101^ An important aspect of MPSs is that they should be “agnostic” to the type of product injected, thereby allowing for the testing of different types of vaccine formulations. Indeed, while we discussed data on the testing of conventional vaccines above, a recent paper demonstrated the ability to assess mRNA vaccines via the potent amplification of antigen-specific memory B cells and neutralizing antibody production in a lymphoid organ-chip. Importantly, they tested COVID mRNA vectored by lipid nanoparticles, demonstrating the versatility of MPSs and suggesting that MPS devices will also be useful tools to assess responses to next-generation mRNA vaccines.^102^

**Figure 3.**
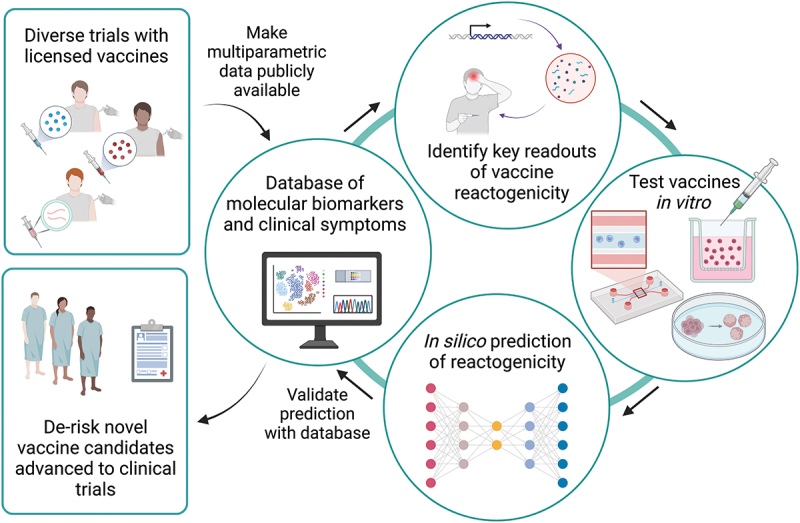
A publicly available database of molecular biomarkers and clinical symptoms produced in humans after vaccination could be used in an iterative process to improve vaccine testing *in vitro*. *in silico* predictions of reactogenicity from *in vitro* data could be validated with the database and used to improve candidates before advancement to clinical trials, thereby derisking the pipeline and reducing costs overall.

Multiparametric data on the reactogenicity of different types of vaccines, including different molecular biomarkers and clinical symptoms, are now required to be made publicly available by vaccine manufacturers in order to improve the analytical capacity of these devices and in particular to predict elevated risks of reactogenicity in individuals and certain cohorts (Figure 3). Indeed, the implementation of MPSs in drug and vaccine development at the preclinical level could drastically derisk the products that get pushed to later clinical trial phases and thereby reduce costs of vaccine development overall as over two-thirds of the costs of vaccine development occurs at the later clinical trials phases.^103^ If MPSs could better elucidate which vaccines elicit the appropriate innate immune response and longer-term adaptive immune protection, while also not inducing harmful inflammatory reactions, this could be a major cost-saving implementation to incorporate in target development pipelines.
